# The Optimal Solution of a Non-Convex State-Dependent LQR Problem and Its Applications

**DOI:** 10.1371/journal.pone.0094925

**Published:** 2014-04-18

**Authors:** Xudan Xu, J. Jim Zhu, Ping Zhang

**Affiliations:** 1 School of Automation Science and Electrical Engineering, Beihang University, Beijing, China; 2 School of Electrical Engineering and Computer Science, Ohio University, Athens, Ohio, United States of America; University of Catania, Italy

## Abstract

This paper studies a Non-convex State-dependent Linear Quadratic Regulator (NSLQR) problem, in which the control penalty weighting matrix 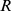 in the performance index is state-dependent. A necessary and sufficient condition for the optimal solution is established with a rigorous proof by Euler-Lagrange Equation. It is found that the optimal solution of the NSLQR problem can be obtained by solving a Pseudo-Differential-Riccati-Equation (PDRE) simultaneously with the closed-loop system equation. A Comparison Theorem for the PDRE is given to facilitate solution methods for the PDRE. A linear time-variant system is employed as an example in simulation to verify the proposed optimal solution. As a non-trivial application, a goal pursuit process in psychology is modeled as a NSLQR problem and two typical goal pursuit behaviors found in human and animals are reproduced using different control weighting 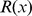. It is found that these two behaviors save control energy and cause less stress over Conventional Control Behavior typified by the LQR control with a constant control weighting 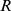, in situations where only the goal discrepancy at the terminal time is of concern, such as in Marathon races and target hitting missions.

## Introduction

### 1.1 Problem Definition

In this paper, we seek an optimal control law 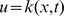, for which the performance index
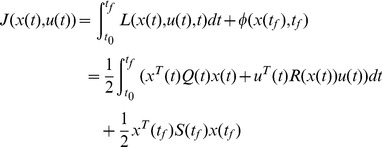
(1)is minimized along the associated closed-loop system trajectory of the Linear Time-variant (LTV) system
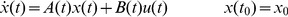
(2)where 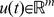 is the control input, 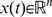 is the system state, 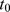 is the starting time, 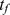 is the terminal time and 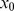 is the initial value of 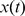 at time 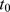. The coefficients 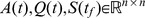, 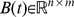, 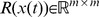. To simplify notation, the dependence of variables on 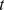 is omitted when no confusion will be introduced in the rest of the paper. It is assumed that 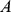, 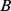, 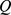 are continuous in 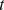, 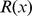 is differentiable with respect to 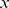, and 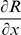 is bounded. The coefficients 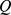 and 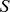 are positive semi-definite symmetric matrices for all 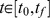, and 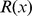 is a positive definite symmetric matrix for all 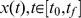. Additional conditions on 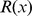 will be imposed in order to obtain the sufficiency for optimality.

It is noted that when the state-dependent matrix 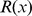 in Eq (1) is replaced by a time-dependent matrix 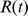, the performance index 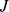 is quadratic and convex in both 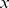 and 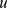, and Eq (1) and (2) constitute the standard Linear Quadratic Regulator (LQR) problem. The classical LQR theory provides a mature way to find an optimal control law for such a convex quadratic performance index. However, the state-dependent coefficient 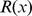 in Eq (1) renders the performance index in the problem no longer convex in both 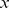 and 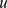, which makes the LQR theory inapplicable here. However, the formalism of the LQR theory is still useful. Therefore, we denote the problem defined above as a Non-convex State-dependent LQR (NSLQR) problem. The associated Riccati Equation of the NSLQR problem is named as Pseudo-Differential-Riccati-Equation (PDRE). In this paper, a necessary and sufficient condition for the optimal solution of the defined NSLQR problem is presented, with an additional condition on 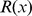, and the optimality of the solution is proven with Euler-Lagrange Equation. The PDRE is also studied to obtain the optimal solution and a theorem is given to estimate the solution of the PDRE.

### 1.2 Related Work

A similar problem has been studied in the context of State-Dependent Riccati Equation (SDRE) control strategy since mid-90's. The strategy, proposed by Pearson[1] and expanded by Wernli and Cook[2], was independently studied by Mracek and Cloutier[3] in 1998. Friedland[4], Salamci and Gokbilen[5], Cimen et.al[6], [7] also contributed to the existence of solutions as well as the properties of optimality and stability. In the SDRE strategy, a nonlinear system is “factored” into the product of the state vector and the state-dependent matrix-valued function in the form
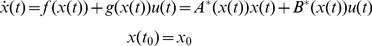
(3)which is a linear structure having state-dependent coefficients. Borrowing the LQR theory, the SDRE strategy postulates an approximately optimal feedback control law as
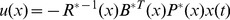
(4)for the performance index
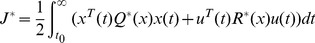
(5)where 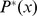 is the solution of an algebraic Riccati Equation (RE) as
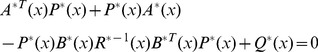
(6)This strategy has been applied in a wide variety of nonlinear control applications, such as autopilot design [8], [9], integrated guidance and control design[10], etc.

However, only the necessary condition for optimality has been studied in the SDRE control strategy, and it cannot always be established. So the optimality of the control law in the SDRE control strategy cannot be guaranteed. Since a simplified algebraic RE is employed to obtain 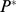 instead of a differential RE, the application of the SDRE control strategy is limited to Slowly Time-Varying and Weakly State-dependent Systems. Moreover, even though the SDRE strategy has been used in many applications, in most cases, the coefficients 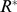 and 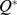 in the performance index 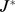 are constant instead of state-dependent, as shown in its formulation[7].

The NSLQR problem defined in this paper focuses on the state-dependent 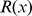 and the time-dependent 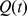 in the performance index and starts with the LTV systems. The optimality of the solution is validated by a rigorous proof with Euler-Lagrange Equation. The solution can be obtained by solving a PDRE associated with the problem. The work is a special case of the SDRE control strategy, but with rigorous mathematical proof. It could be considered as a theoretical support for the SDRE control strategy.

On another aspect, the solution of the optimal LTV problem is usually obtained through numerical approximation approaches, which can be roughly classified into offline and online methods. The offline method usually pre-computes solutions and stores them for fast online look-up [11], [12]. Since the computation grows exponentially with the size of the control problem, offline methods are normally used in small- and medium-size applications. The most prominent online methods are active set[13] and interior point method[14]. The method of active set performs well in large-size cases even though its convergence rate is unknown. For the interior point method, the reported lower iteration number is larger than the practically observable number. In Ref. [15] and [16], a fast gradient method is introduced to help calculating the lower iteration bound for a quadratic LTV optimal problem with input constraints. Though the work listed above is mainly about the optimal problem with a time-dependent 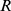, the formalism is still applicable when developing the numerical solution for the defined NSLQR problem.

### 1.3 Application Background

The NSLQR problem discussed in this paper can be applied to model a psychological goal pursuit process, as a non-trivial example.

**Figure 1:**
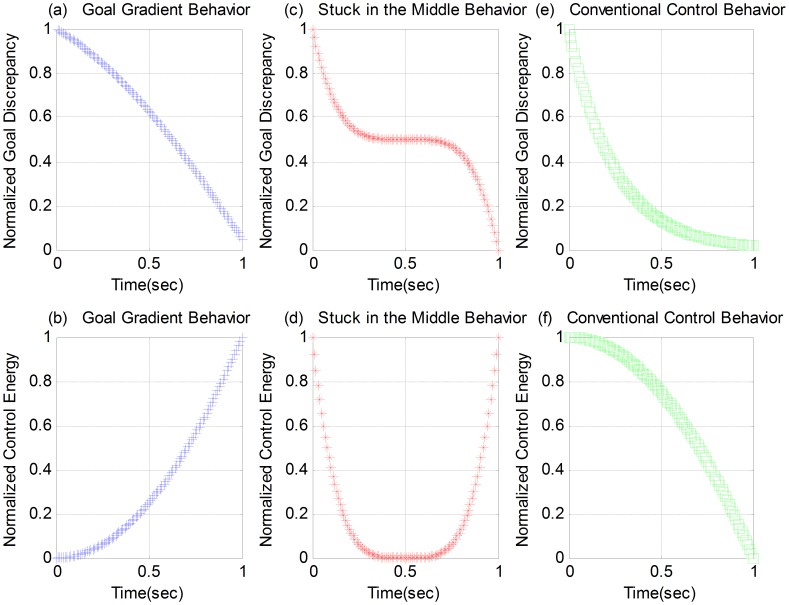
Three Different Behaviors: GGB, SMB and CCB.

Psychologists observe that there are two different behaviors when intelligent creatures pursue a goal. One is the Goal-Gradient Behavior (GGB) [17]–[20], in which the control effort to reach a goal increases monotonically with the proximity to the desired end state, such as the predator stalking behavior and the deadline beating behavior. Fig. 1 (a) and (b) give the normalized goal discrepancy and control effort of the GGB. As it is shown, with a monotonically increasing control energy, as the goal is approached the discrepancy reaches zero faster at the end of the process than it does at the beginning. The other is the Stuck-in-the-Middle Behavior (SMB) [21], in which the control effort to reach a goal is high at the beginning of the goal pursuit and when the desired end state is in sight, but it is maintained at a low level in between, such as what athletes do in Marathon. Part (c) and (d) in Fig. 1 show typically the SMB where the goal discrepancy decreases faster at the two ends than it is in the middle of the goal pursuit process and the control effort is maintained at a low level in the middle.

Both the GGB and the SMB are different from the Conventional Control Behavior (CCB) found in an engineering control system, as shown in part (e) and (f) in Fig. 1. For the CCB, the control effort is proportional to the goal discrepancy, so the effort decreases with proximity to the desired end. The purpose of this paper is to study which one pf these three behaviors is the best. Some computational models of the GGB have been proposed based on psychological interpretation [22], [23]. In this paper, a single-task goal pursuit process is modeled as a NSLQR problem and the three behaviors are reproduced for comparison, facilitating “a deeper understanding of mathematical characterizations of principles of adaptive intelligence”[24] instead of psychological interpretation.

In the sequel, Section 2 presents the necessary and sufficient condition for the optimality of the solution to the NSLQR problem. Section 3 analyzes the solution of a PDRE involved in the NSLQR problem and presents a Comparison Theorem. Section 4 verifies the feasibility of the NSLQR theory with a LTV system and applies the NSLQR to model a goal pursuit process. The numerical simulation results are presented to demonstrate that the GGB and SMB save control energy and cause less stress over the CCB in some applications. Conclusion and Future Work are presented in Section 5.

## Analysis of the Optimality of the Solution

In this section, the main result of this paper is presented: the necessary and sufficient condition of the optimality of the solution to the NSLQR problem defined in Eq (1) and (2). Before that, an Optimality Lemma is introduced first. The Optimality Lemma discusses a more general optimal problem, compared with the NSLQR problem. To differ from the performance index 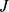 in the NSLQR, we denote the performance index in the Lemma as 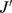 with an associated general 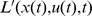. The associated augmented performance index is defined as 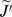 in Eq (10), to distinguish it from the 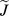 in the NSLQR problem. In this paper, the header “
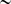
” indicates the associated augmented performance index, as presented on Page 379 in Ref [25].

**Lemma 1** *For the problem of finding an optimal control law* 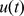 *, for which the performance index*

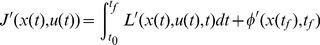
(7)
*is minimized along the associated closed-loop system trajectory* 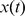 *of the LTV system (2), with a fixed starting time* 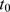 *and terminal time* 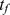 *, to simplify notation, define*

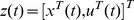
(8)

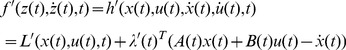
(9)
*and an augmented performance index as*

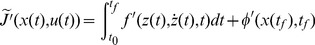
(10)
*where* 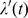 *is the Euler-Lagrange multiplier, with the boundary condition as*

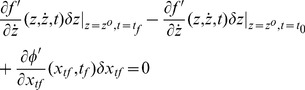
(11)
*Then the point* 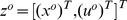 *that satisfies Euler-Lagrange Equation*

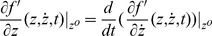
(12)
*is the optimal solution if the augmented performance index* 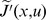 *is strictly convex in* 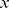 *, uniformly in* 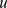 *; and strictly convex in* 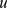 *, uniformly in* 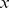 *.*

Here, the superscript “
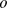
” indicates the optimal solution.

**Proof 1** *It can be proven that* 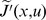 *is equivalent to* 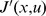 *in that they have the same minimizing function, if it exits* [*25*] *. With Euler-Lagrange* *Equation (12* *), the variation of* 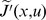 *at* 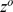 *can be written as*

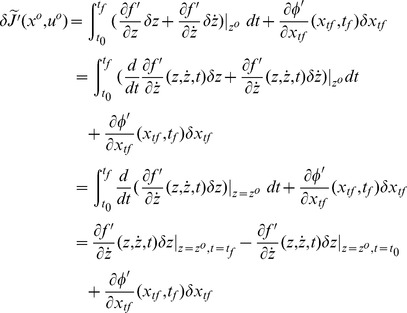
(13)
*To make the point* 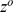 *be the optimal solution, the variation* 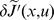 *is supposed to be* 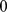 *at* 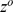 *, which leads to Eq (11). However, the point* 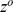 *satisfying Eq (11) and (12) can either an extreme point or a saddle point of the* 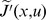 *. Now we prove that, under the convexity constraint stated in Lemma 1, the point* 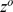 *is a minimum point by contradiction.*

*With* 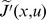 *being strictly convex in* 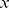 *, uniformly in* 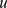 *, it follows that* 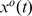 *satisfying Eq (12) is a minimum point of* 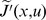 *with respect to* 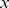 *, uniformly in* 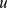 *. Then we have*

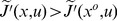
(14)
*for every* 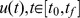 *and every* 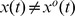 *. Similarly, we have*

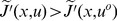
(15)
*for every* 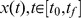 *and every* 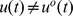 *.*

*Assume that the point* 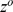 *is a maximum point of* 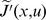 *. Then since* 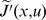 *is continuous, there exists a* 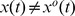 *, such that*

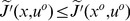
(16)
*which contradicts Eq (14). Thus* 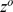 *cannot be a maximum point of* 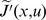 *. Now assume that* 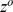 *is a saddle point of* 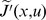 *. Then by continuity of* 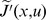 *, there must be a* 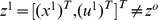 *and a* 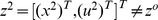 *such that*

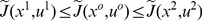
(17)
*Then with Eq (15), we have*

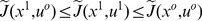
(18)
*The equality holds if and only if* 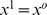 *and* 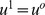 *, which contradicts the defination of* 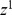 *. The inequality contradicts Eq (14). Thus the point* 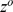 *cannot be a saddle point of* 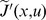 *either. Then the point* 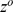 *must be a minimum point of* 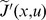. *So the solution* 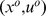 *minimizes the performance index* 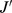 *in (7).*

From the proof above, it can be said that the classical LQR is a special case of Lemma 1. In the classical LQR theory, the sufficiency of the optimality of the solution obtained from Euler-Lagrange Equation is guaranteed by the convexity of the augmented performance index 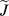 in its arguments 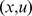 [25]. However, in the NSLQR, 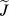 is no longer convex in 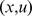 because of the state-dependent 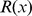. The theorem below shows that the solution of Euler-Lagrange Equation is still optimal for the NSLQR problem with a constraint on 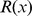.

**Theorem 1** *Under the convexity constraint that the function* 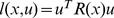 *is a strictly convex function in* 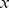 *, uniformly in* 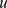 *, the state feedback control law*

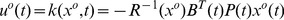
(19)
*for the NSLQR problem defined in Eq (1) and (2), and the associated closed-loop system trajectory* 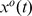 *as*

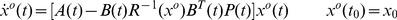
(20)
*minimizes the performance index (1) if and only if the* 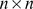 *matrix* 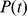 *satisfies the PDRE as*

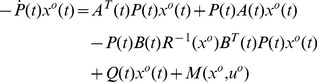
(21)
*with*

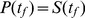
(22)
*where the column vector*

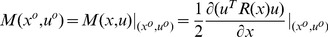
(23)


Here, the explicit 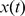 on both sides of the equation can be eliminated for some sharpened 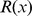. One example is discussed in Section 3.

This theorem provides an optimal solution, which is similar to that of the classical LQR theory, for the NSLQR problem. However, the PDRE (21) is with an additional term “
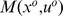
”, compared with the standard Riccati Equation in the LQR theory. This term comes from the derivative of the state-dependent 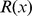 with respect to 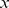 in the Euler-Lagrange Equation, as detailed in Proof 2. Theorem 1 can be proven with Lemma 1 as follows.

**Proof 2** *To simplify notation, define*

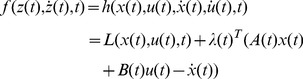
(24)
*where*

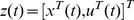
(25)
*and* 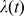 *is the Euler-Lagrange multiplier. The admissible directions of* 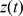 *are denoted as*

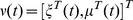
(26)
*with* 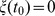 *,* 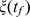 *,* 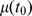 *, and* 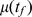 *being free, since the initial value of* 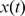 *is fixed. The augmented performance index is*

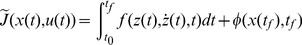
(27)
*Similarly,* 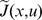 *is equivalent to* 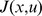 *in that they have the same minimizing function, if it exits* [*25*] *. Now we need to prove that* 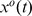 *,* 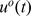 *defined in Eq (19) and (20) minimizes the augmented performance index (27) if and only if* 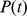 *satisfies the PDRE (21) and* 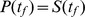 *.*

*We start with the necessary condition. From Euler-Lagrange Equation, it is known that for a point* 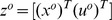 *to be an optimal solution, the necessary condition is*

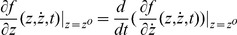
(28)
*with a boundary condition, as discussed later. For a state-dependent* 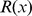


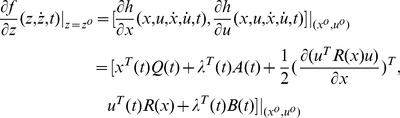
(29)

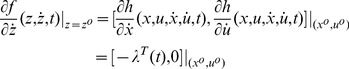
(30)
*To simplify notation, the column vector* 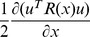 *is denoted as* 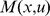 *. Substituting the two equations above into Eq (28), we have*

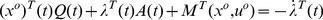
(31)

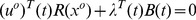
(32)
*which leads to a control law as*

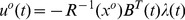
(33)
*with* 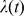 *satisfying*

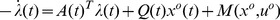
(34)
*The variation of* 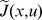 *at the point* 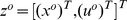 *can be written as*

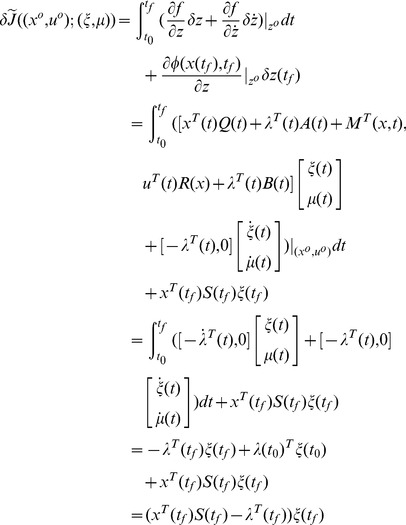
(35)
*To minimize* 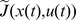 *, we need to achieve* 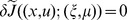 *for all admissible directions. Since* 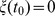 *and* 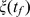 *is free, the terminal value of* 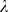 *needs to satisfy*

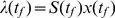
(36)
*We choose* 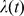 *such that it is linearly related to* 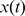 *through* 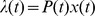 *. Then the boundary condition Eq (36) becomes*

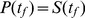
(37)
*Substituting the assumption* 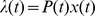 *into Eq (33) and (34), we obtain a control law as*

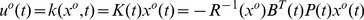
(38)
*where* 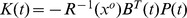 *, with the associated closed-loop system as*

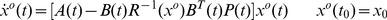
(39)
*where* 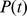 *satisfies *

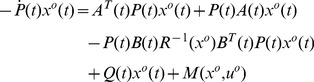
(40)
*and* 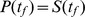 *.*

*Now we prove that the solution* 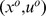 *from the necessary condition of optimality also satisfies the sufficiency condition using Lemma 1.*

*First, it is easy to verify that the solution* 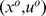 *in Eq (38) and (39) with* 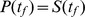 *satisfies Eq (11) and (12) in Lemma 1. Now we prove that* 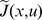 *in Theory 1 is strictly convex in* 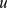 *, uniformly in* 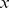 *. Considering an arbitrary fixed* 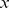 *, we set* 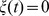 *, then the variation of* 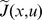 *in* 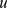 *is*

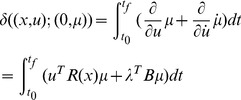
(41)In terms of Eq (41), we have
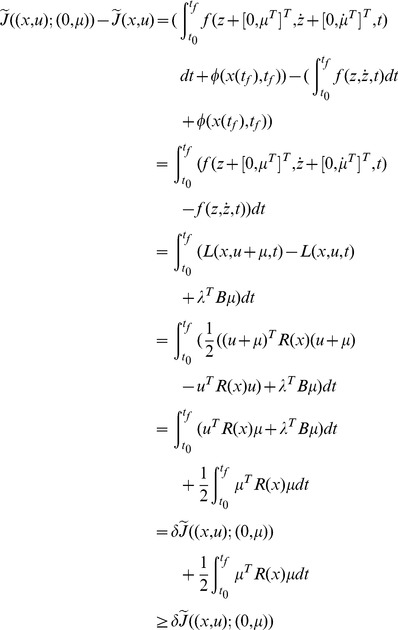
(42)
*Since* 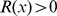 *, the equality holds when and only when* 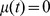 *. So* 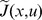 *is strictly convex in* 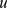 *, uniformly in* 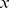 *.*

*Now we prove that* 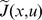 *is strictly convex in* 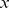 *, uniformly in* 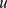 *. Setting* 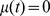 *, the variation of* 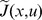 *in* 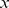 *is written as*

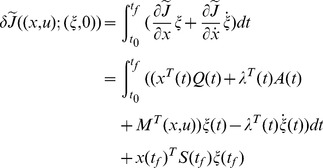
(43)
*where* 
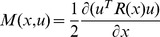
 *. In term of Eq (43), we obtain*

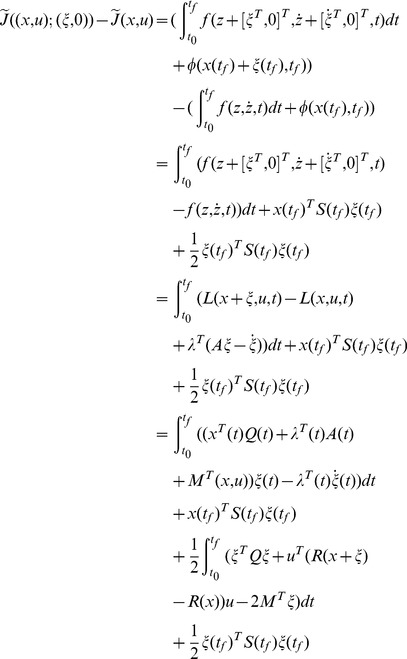
(44)
*Since the function* 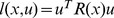 *is a strictly convex function in* 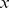 *, uniformly in* 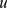 *as it is stated in Theorem 1, we have*

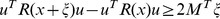
(45)
*Then*

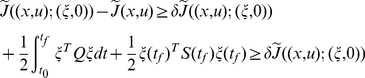
(46)
*the equality holds when and only when* 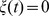 *. So* 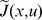 *is strictly convex in* 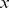 *, uniformly in* 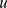 *.*

*From the analysis above, it holds that the* 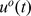 *and* 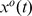 *defined in (19) and (20) with the boundary condition (22) satisfy (11) and (12) in Lemma 1. In addition, the augmented performance index* 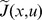 *is strictly convex in* 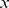 *,* 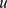 *separately, uniformly in the other one. We conclude that* 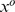 *and* 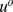 *minimize the performance index* 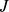 *in Eq (1) for the NSLQR problem. So* 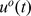 *defined in (19) is the optimal control law for the NSLQR problem.*

From the proof of Theorem 1 and Lemma 1, we obtain a corollary for the defined NSLQR problem with a general 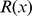 as following.

**Corollary 1** *For the optimal problem defined in (1) and (2) with* 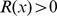 *and free boundary values of* 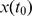 *and* 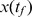 *, the* 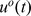 *and* 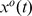 *defined in (19) and (20) with* 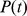 *satisfying (21) and (22) is either a minimum or a saddle for the performance index* 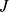 *in (1).*

It follows from Eq (42) that 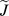 is strictly convex in 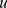, uniformly in 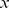 if 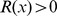. It then follows from the proof of Lemma 1 that 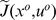 cannot be a maximum. Detailed proof is omitted.

***Remark***: The corollary signifies that the optimal control law (19) gives the minimum cost for a particular 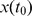 among all possible control laws for a general 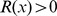, since 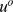 is the minimum point of 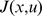 with respect to 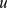. However, the cost may be lowered if a different 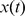 trajectory with a different 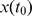 is chosen since 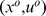 can be a saddle of 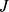. So for a given 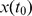, the control law (19) gives the optimal solution.

From the analysis, it can be said that, for the NSLQR problem with a general 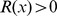, the optimal solution 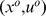 needs to be evaluated for every specific 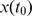. Whereas in the classical LQR problem or the SDRE problem, the optimal solution can be explicitly written as a function of 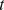 or 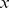, uniformly for any 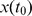. For the NSLQR to have such uniform solutions, 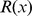 has to satisfy the additional constraint that the function 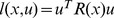 is strictly convex in 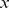, uniformly in 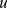.

## Analysis of the Solution for PDRE

### 3.1 A Sharpened 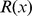


The PDRE (21) of the NSLQR problem is different from the RE in the SDRE control strategy literature in

1. There is an additional term 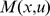 in the PDRE (21), which is derived from the derivative of the state-dependent 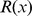 with respect to 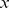;
2. It is a differential RE instead of an algebraic RE;
3. The system state 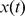 appears on both sides of the equation.

The way to solve the SDRE is not applicable for the PDRE. To obtain the optimal solution of the NSLQR, it is necessary to investigate the solution of the PDRE (21). In this section, a sharpened 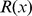 is studied as an example.

As stated in Theorem 1, the function 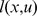 needs to be strictly convex in 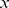, a quadratic function of 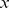 seems to be a reasonable choice for the matrix 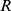. So consider
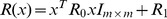
(47)where 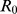 is a 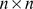 symmetric, positive semi-definite constant matrix, 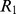 is a 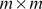 symmetric, positive definite constant matrix and 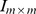 is the 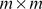 identity matrix. The term 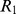 guarantees that 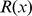 is invertible. Then
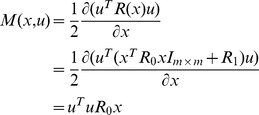
(48)and the PDRE (21) becomes
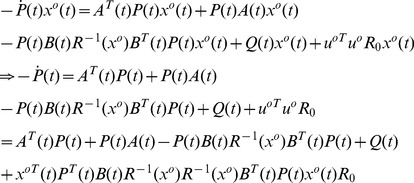
(49)which can be denoted as
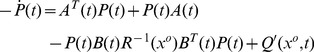
(50)where 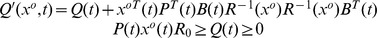, and 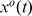 satisfies the closed-loop system (20).

***Remark***: The PDRE (50) is coupled with closed-loop system (20). In classical LQR theory, system state 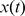 and the solution of Riccati Equation 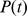 can be obtained through a 2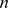-dimensional Hamiltonian matrix [25] or by decoupling the system plant and the Riccati Equation. However, in the NSLQR problem, the 2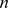-dimensional Hamiltonian matrix is not linear any more, and the decoupling is not applicable. The PDRE (50) has to be solved together with the closed-loop system (20).

To generalize the results, the PDRE (50) can be rewritten into a general form as:
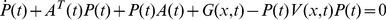
(51)with a given terminal value 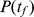, and 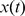 is a continuous single-valued function of 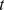. The matrices 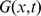, 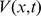 are positive semi-definite, symmetric, and continuous in both arguments.

For convenience of reference, the PDRE (51) is denoted as 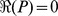 in the sequel. The dependence of the variables on 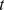 is omitted, for example, 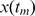 is denoted as 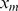. The time argument of matrices and vectors are omitted when no misunderstanding is introduced. For instance, 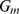 denotes the value of matrix 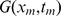.

### 3.2 A Comparison Theorem for the PDRE

The propositions and theorem introduced below are derived from Proposition 7 and 8 in [26]. In [26], similar results are developed for time-dependent Riccati Equations with initial values. Now, a Comparison Theorem for the PDRE (51) with a terminal value is given. Before presenting the theorem, four propositions need to be established first.

**Proposition 1** *Let* 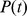 *be a symmetric solution of the PDRE (51) on* 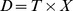 *, where* 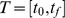 *,* 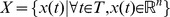 *.*

1. If 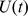 is a symmetric solution of the inequality 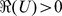 on 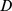 such that 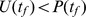, then 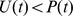 on 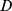.
2. If 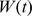 is a symmetric solution of the inequality 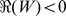 on 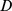 such that 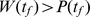, then 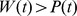 on 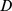.

**Proof 3** *Since the matrices and vectors in the PDRE (51) are continuous in the arguments,* 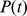 *,* 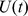 *and* 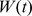 *are continuous in* 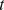 *. We start with part (1) of the proposition.*

**Figure 2:**
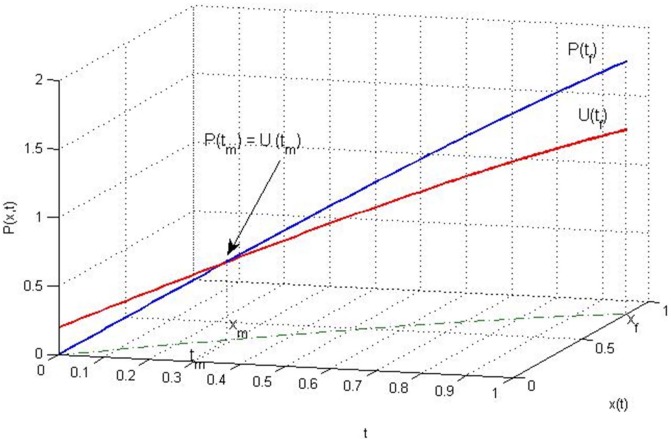
Trajectories of 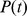 *and* 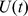.



Suppose that 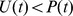 does not hold on 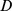, there must be a time 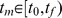 by the Mean Value Theorem, such that
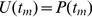
(52)and
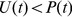
(53)for any 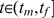, as shown in Fig. 2. Let

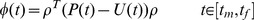
(54)where 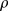 is a non-zero constant vector. Then
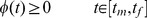
(55)and the equality holds if and only if 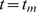.
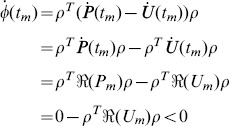
(56)Therefore, 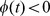 in a right neighborhood of 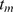, which contradicts Eq (55). So 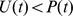 holds on 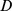.

Part (2) can be proven similarly.

**Proposition 2** *Let* 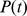 *be a symmetric solution of the PDRE (51) on* 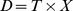 *, where* 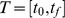 *,* 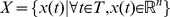 *.*

1. If 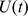 is a symmetric solution of the inequality 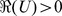 on 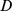 such that 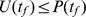, then 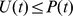 on 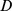.
2. If 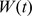 is a symmetric solution of the inequality 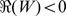 on 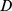 such that 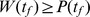, then 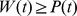 on 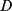.

**Proof 4** *The inequality part of the proposition has been proven in Proposition 1. The following part is the proof for the case in which* 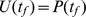 *. We start with part (1).*

As it is discussed in Proof 3, 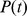, 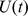 and 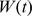 are continuous in 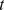. Assume that 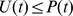 does not hold on 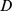 when 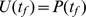, there must be a period 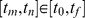, such that
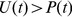
(57)for any 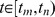, and
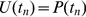
(58)Let
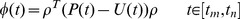
(59)where 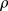 is a non-zero constant vector. Then
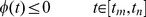
(60)and the equality holds if and only if 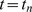.
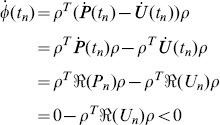
(61)Therefore, 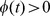 in a left neighborhood of 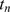, which contradicts Eq (60). So 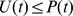 holds on 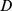.

Part (2) can be proven similarly.

**Proposition 3** *Let* 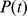 *be a symmetric solution of the PDRE (51) on* 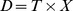 *, where* 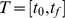 *,* 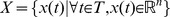 *.*

1. If 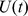 is a symmetric solution of the inequality 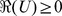 on 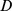 such that 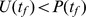, then 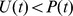 on 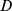.
2. If 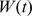 is a symmetric solution of the inequality 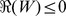 on 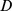 such that 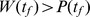, then 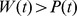 on 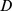.

**Proposition 4** *Let* 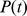 *be a symmetric solution of the PDRE (51) on* 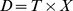 *, where* 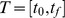 *,* 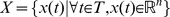 *.*

1. If 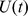 is a symmetric solution of the inequality 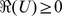 on 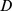 such that 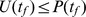, then 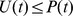 on 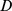.
2. If 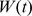 is a symmetric solution of the inequality 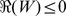 on 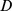 such that 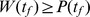, then 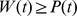 on 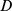.

The proofs of these two propositions are similar to those of Propositions 1 and 2.

These four propositions give a boundary estimation for the solution of the PDRE. Based on the four propositions discussed above, a Comparison Theorem is introduced for the PDRE (51).

**Theorem 2** *For* 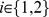 *, let* 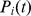 *be the solution of the PDRE*

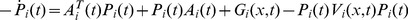
(62)
*on* 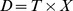 *, where* 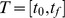 *,* 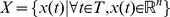 *. If* 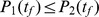 *, and*

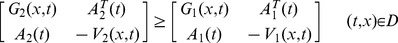
(63)
*Then* 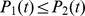 *on* 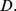 *.*

**Proof 5** *Let* 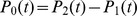 *, then* 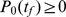 *.*

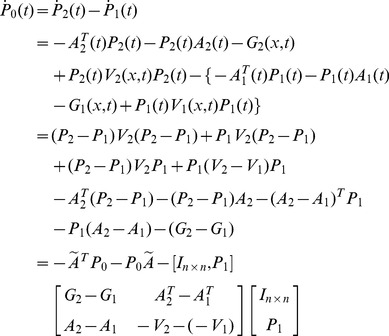
(64)where 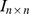 *is the* 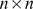 *identity matrix and* 
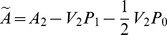
 *.*

Since
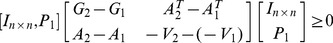
(65)
*for the PDRE* 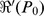 *defined as*

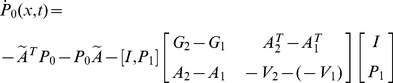
(66)
*we have* 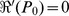 *,* 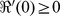 *,* 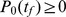 *. It is readily concluded that* 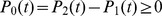 *for all* 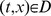 *by Proposition 4. So* 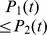 *on* 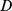 *.*

### 3.3 Application of Comparison Theorem

For the PDRE (50), it is readily verified that 
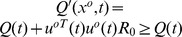
 and 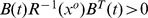. Then we have
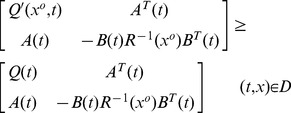
(67)and
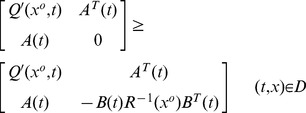
(68)With Theorem 2 in Section 3.2, it follows that the solution 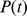 of the PDRE (50) satisfies
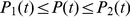
(69)where 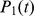 is the solution of
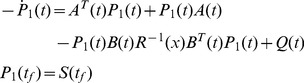
(70)and 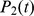 is the solution of
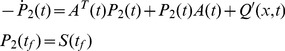
(71)These two equations are differential REs, which are similar to the algebraic RE in the SDRE control strategy. The analysis shows that the solution of a PDRE can be estimated by the solutions of two differential REs. Thus the methods of solving a differential RE can be borrowed to facilitate the solution to a PDRE, such as determining the initial value for 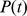.

## Simulation

In this section, the NSLQR problem studied above is applied to two specific simulation cases to first verify the optimality numerically and then to model the goal pursuit process introduced in the section Introduction, so that the three goal pursuit behaviors, GGB, SMB and CCB are reproduced for further studying.

The NSLQR problem is, technically, a Two-Point Boundary Value (TPBV) Problem, since the initial value of the system plant (20) 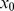 and the terminal value of the PDRE (21) 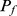 are known. A shooting method is employed in the simulation with sacent iteration to solve this TPBV problem [27]. The convergence of the method is slightly slower than being second-order with the chosen 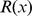, as it is discussed in [28] and [29]. The solver of “ode4 Runge-Kutta” is chosen in the simulation with a fixed step of 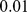 second. The simulation error threshold is set as 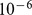.

### 4.1 Numerical Verification of the NSLQR Optimality

#### 4.1.1 Simulation Model

In this section, we consider a specific optimal problem of seeking a control law 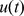 to minimize the given performance index
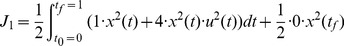
(72)along the first-order LTV system as
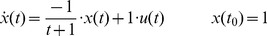
(73)with 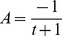, 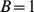, 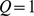, 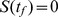 and 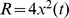.

**Figure 3:**
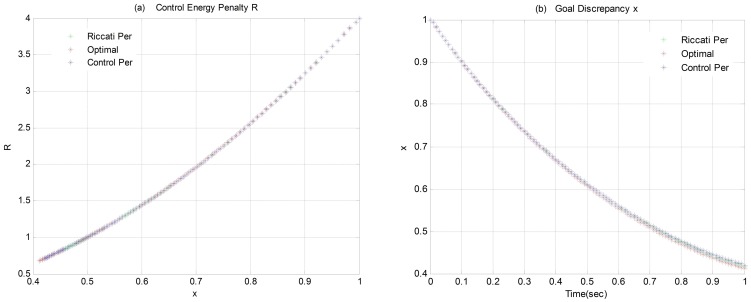
System Behavior of Simulation Case 01: a) Control Energy Penalty R vs. Goal Discrepancy x; b) Goal Discrepancy x.

Three forms of control law are considered. The first one is the optimal control law 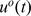 defined in Eq (19) with the 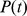 from the PDRE (21). The second control law is the same 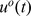 in Eq (19) but with the 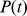 from a standard Differential Riccati Equation, which has no additional term of 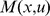. The third control law is the same 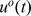 in Eq (19) with a perturbation of 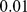. To differentiate with each other, the three control laws are named as “Optimal Solution”,“Riccati Perturbation” and “Control Perturbation”, respectively. Table 1 summarizes the parameter details used in this simulation case as well as the values of performance index 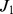. Fig. 3 gives the system behaviors with the three different control laws.

**Table 1:** The Values of Parameters in Simulation Case 01.

	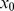	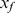	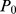	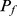	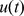	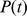	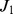
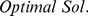	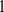	0.413	0.5	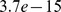	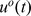	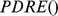	0.237653
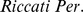	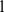	0.417	0.5	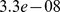	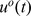	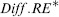	0.237672
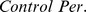	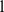	0.421	0.5	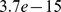	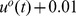	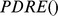	0.237695

*Diff. RE: 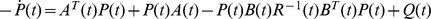.

#### 4.1.2 Discussion of Results

From Fig. 3, it is valid to say that the three control laws, with the same function of 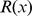, all bring the system state a stable behavior. However, the optimal control law 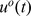 supplies the minimal performance index 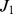, as shown in Table 1. Even though it cannot be said with great confidence that the control law 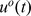 is optimal since it is difficult to verify infinite numerical examples. It is still proved that the classical LQR solution, which is the “Riccati Perturbation” case, provides a greater value of 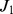 than the optimal control law 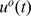 does, so the classical LQR theory is not applicable to the NSLQR problem anymore. Moreover, the NSLQR theory does provide the minimal value of 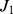 among all three control laws, which verifies the optimality of the NSLQR theory at some degree.

### 4.2 Application of the NSLQR to Goal Pursuit Processes

#### 4.2.1 Modeling Goal Pursuit Behaviors

From a psychological perspective, the system state 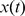 in the NSLQR problem represents the *goal discrepancy*. The parameter 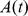 in system model (2) represents the *goal attraction*. For a constant 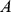, all the eigenvalues having negative real parts (asymptotically stable) means the goal is attractive; if the eigenvalues are with non-positive real parts and those eigenvalues with zero real parts are simple (marginal stable), then the goal is neutral; otherwise (unstable), the goal is repulsive. Similar interpretations apply to time-varying 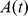, where the asymptotic stability, marginal stability and instability can be interpreted as attractive, neutral and repulsive goals. The input 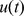 represents the level of *control effort*, while the parameter 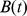 is treated as *control effectiveness*. In the performance index 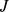, the weighting coefficient 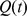 functions as *goal discrepancy penalty*. A greater value of 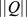 results in less discrepancy; and the weighting coefficient 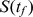 is known as *terminal penalty*. A greater value of 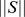 leads to smaller terminal goal discrepancy; the weighting coefficient 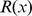 is *control energy penalty*, which depends on goal discrepancy. A greater value of 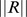 means less control energy expenditure is allowed.

#### 4.2.2 Simulation Model

**Figure 4:**
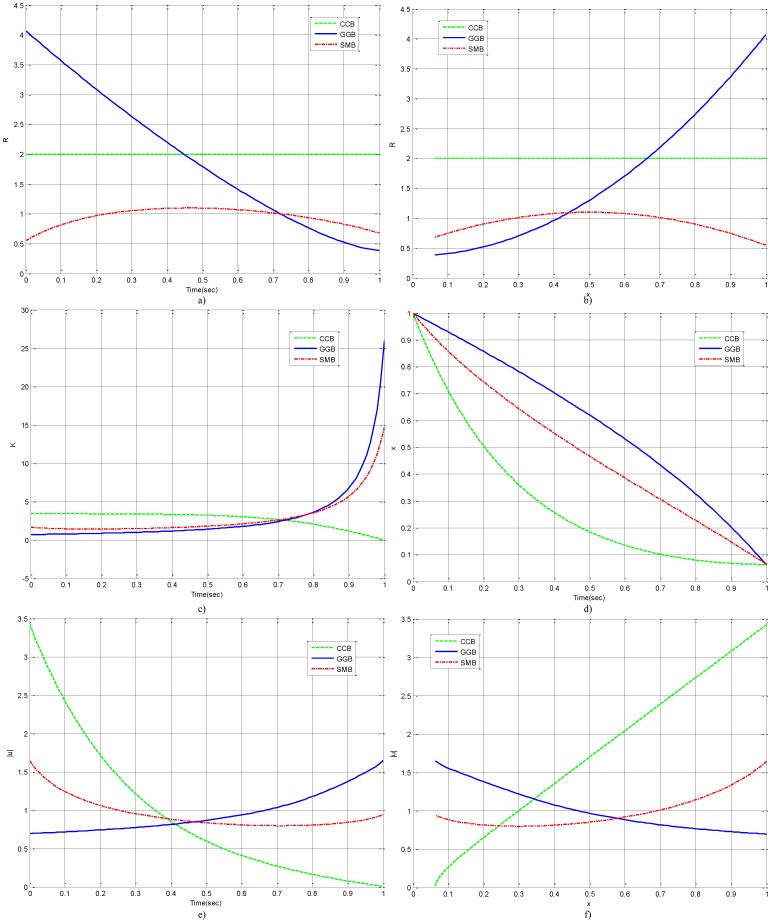
Three Goal Pursuit Behaviors: a) Control Energy Penalty R vs. Time; b) Control Energy Penalty R vs. Goal Discrepancy x; c) Feedback Gain K; d) Goal Discrepancy x; e) Control Effort u vs. Time; f) Control Effort u vs. Goal Discrepancy x.

As an initial study, we consider a first-order linear goal attainment process in the simulation case. The process is set as
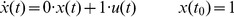
(74)which is a neutrally attractive goal process. The parameter 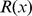 is the concern in the current simulation case study. Based on the analysis above, we hypothesize that the GGB can be produced by 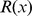 that is a monotonically increasing function of 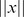; the SMB can be produced by 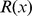 that is a hump function of 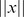; and the CCB can be produced by a constant 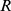. Table 2 gives the value of the parameters used in the simulation. The simulation results are presented in Table 3 and Fig. 4. From Table 2, it can be seen that the choice of 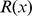 in the GGB satisfies the convexity constraint of the function 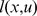, so the solution is optimal for the GGB. Even though the convexity constraint is not satisfied in the SMB, from Corollary 1, it still can be said that the solution is optimal since 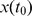 is fixed. For a meaningful comparison, the parameters are adjusted such that three behaviors achieve roughly equal terminal values, as the values of 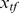 shown in Table 3.

**Table 2:** The Values of Parameters in Simulation Case 02.

	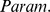	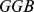	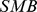	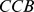
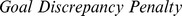	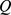	1	1	23.5
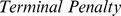	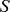	10	10	0
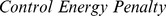	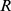	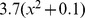	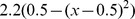	2
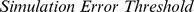	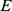	0.000001	0.000001	0.000001
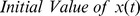	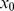	1	1	1
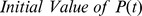	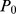	2.831	0.903	6.841

**Table 3:** Simulation Results of Three Goal Pursuit Behaviors.

	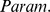	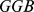	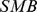	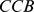
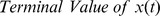	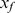	0.064	0.064	0.064
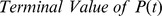	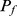	10.000	10.000	0.000
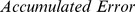	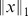	59.847	49.183	29.680
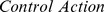	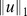	96.937	95.770	95.180
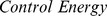	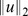	9.977	9.730	13.207
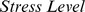	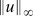	1.651	1.643	3.421

From Fig. 4 Part d), it can be seen that, the NSLQR method has successfully modeled the goal pursuit process with three behaviors. Part a) and Part b) show the control energy penalty matrix 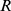 vs time 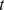 and goal discrepancy 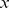, respectively. The hypothesis is validated that when 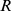 is a monotonically increasing function of 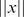, the goal pursuit process can exhibit the GGB; when 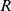 is a hump function of 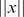, the goal pursuit process can exhibit the SMB; and when 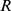 is a constant, the goal pursuit process can exhibit the CCB. Part e) and Part f) present the control effort 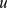 vs time 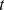 and goal discrepancy 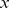, respectively. For the CCB, the control effort increases as it approaches the goal; for the GGB, the control effort decreases with goal discrepancy; and for the SMB, the control effort is higher at two ends than in the middle.

Table 3 lists some selected norms of the goal discrepancy 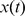, and the control effort 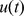. It shows that, with the same initial value 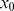 and terminal value 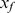, the CCB features the least accumulated error 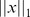, but consumes the most control energy 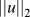 and suffers from the highest stress level 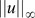. The SMB consumes the least control energy and sufferers from the lowest stress level at the price of a higher accumulated error. The CCB is in between of these two.

#### 4.2.3 Discussion of Results

Based on the simulation results above, it is concluded that in pursuing a goal with a finite terminal time (deadline), the GGB and the SMB behaviors may save control energy and reduce stress level over the CCB. However, the CCB has the least accumulated error. So the GGB and SMB may be beneficial in applications where only the level of goal attainment at the terminal time is of concern, such as a deadline beating process. However, the GGB or SMB would not a preferred choice when the goal needs to be maintained over long time or needs to be approached smoothly.

## Conclusion and Future Work

In this paper, a necessary and sufficient Optimality Theorem with rigorous mathematical proof for the NSLQR problem with a convexity constraint on 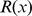 is presented. It is also argued that for given 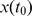, the NSLQR gives an optimal solution. A Comparison Theorem for the solution of the PDRE with a general form for the NSLQR problem is presented as well. In the simulation, the NSLQR is first applied to a first-order LTV system to verify the proposed theory. The NSLQR is then used to model two psychological behaviors (GGB and SMB) in goal pursuit processes identified from psychology, along with the typical behavior of engineering control systems (CCB) by employing different control energy weighting 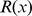. The simulation results show that the NSLQR modeling method can reproduce the three goal pursuit behaviors and the psychological goal pursuit behaviors can be more beneficial than the CCB in terms of energy saving and stress reduction in applications where only the goal discrepancy at the terminal time is of concern, such as in Marathon race, animal stalking, beating a deadline or hitting a target.

In this paper only some scalar cases of the goal pursuit process are studied; studies of the multi-variable cases are the next steps of our work. In the current study, the parameter 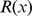 is selected to reproduce the goal pursuit behaviors. Similar results should be achievable with a state-dependent goal discrepancy weighting 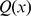, which would be more akin to intuitive psychological tendency to employing the GGB and SMB strategies in terminal goal pursuit processes; whereas the control weighting modeling is more akin to conscious choice of the GGB and SMB for its energy saving and stress reduction benefits. Since for the NSLQR problem the PDRE has to be solved simultaneously with the closed-loop system, it is a TPBV problem. An inherent difficulty in this TPBV problem is how to determine the initial value of 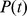. An Approaching-Horizon algorithm based on a shooting method is developed to address this problem, which will be presented in detail in a separate paper.
